# Editorial: Systemic regulation of organ homeostasis and implications of hormones and immunity, Volume II

**DOI:** 10.3389/fendo.2023.1235274

**Published:** 2023-06-26

**Authors:** Vijaya Kumar Pidugu, Tatjana S. Kostic, Silvana A. Andric, Anil M. Tharappel, Taka-Aki Koshimizu

**Affiliations:** ^1^ Laboratory of Cancer Biology and Genetics, Center for Cancer Research, National Cancer Institute, Bethesda, MD, United States; ^2^ Laboratory for Reproductive Endocrinology and Signaling, Laboratory for Chronobiology and Aging, Center of Excellence for Reproductive Endocrinology and Signaling, Faculty of Sciences, University of Novi Sad, Novi Sad, Serbia; ^3^ Department of Pharmacology and Toxicology, R. Ken Coit College of Pharmacy, The University of Arizona, Tucson, AZ, United States; ^4^ Department of Pharmacology, Division of Molecular Pharmacology, Jichi Medical University, Tochigi, Japan

**Keywords:** homeostasis, hormones, endocrine system, hypothyroidism, ISL2A, FSHR, PCOS, colorectal cancer

The multicellularity of an organism enables cells in the body to make complex tissues and organ systems that orchestrate their complex functions. The coordination of specialized functions of some of these cells is to assure the maintenance of basic metabolic functions and requirements of all other surrounding cells for the survival of organs and organ systems of the body. The endocrine system plays a critical role in regulation of homeostasis by executing a vastly complex network of molecular signaling mechanisms that communicate information in the form of hormones released by the cells. The hormone signals, in turn, stimulate organ systems to restore homeostasis. Although endocrine and immune systems regulate distinct functions, the coordinated response of these systems is needed for the maintenance of homeostasis. The altered endocrine system in the body affects the balance between pro-and anti-inflammatory immune responses. If homeostasis is not restored, the imbalance may lead to various diseases.

During the evolution process, multiple positive and negative feedback loops have developed between endocrine and immune systems to coordinate the normal body function of an organism. It is important to study these immune-endocrine interactions to understand the underlying molecular mechanisms that regulate systemic metabolism and disease progression. This could allow us to explore optimal disease management and treatment strategies for patients. In this context, the present Research Topic was intended to study the critical biological interactions of endocrine and immune systems to understand the pathophysiology of diseases and possible treatment options. Individually and collectively, the research and review articles collected on this Research Topic make a significant addition to our understanding of immune-endocrine interactions in the context of endocrine related diseases.

The ISL LIM homeobox 2a protein (ISL2A), a novel transcription factor has been shown as a regulator of early angiogenesis, exocrine pancreas development, and neuron differentiation. However, the functional role of ISL2A in the hypothalamus-pituitary thyroid axis is elusive. Yan et al. have established homozygous *isl2a* knockout zebrafish using CRISPR/Cas9 system. Molecular characterization of isl*2a* mutant zebrafish revealed a novel transcriptional regulatory role of *isl2a* in pituitary cell differentiation. This study should facilitate future studies to unravel the molecular mechanism of hypothyroidism and possible drug development in this zebrafish model. Stojiljković et al. developed an extended, stoichiometric model of the hypothalamic-pituitary-adrenal (HPA) axis for better understanding individual or combined effect of corticotrophin-releasing hormone (CRH) and arginine vasopressin (AVP) on the secretion of adrenocorticotropic hormone (ACTH) by corticotropic cells in the human pituitary gland. The extended HPA model is associated with previous experimental findings and highlights possible implication for future studies related to homeostasis dynamic crisis, autoimmune inflammation, and exogenous administered AVP treatment. Gentil et al. conducted a randomized clinical trial on type 2 diabetes mellitus (T2DM) patients to evaluate the beneficial effect of three different aerobic exercise protocols such as moderate-intensity continuous training (MICT), short-interval high-intensity training (S-HIIT), and long interval high-intensity training (L-HIIT). Parallel comparison of cardiometabolic variables showed fundamental differences among T2DM patients undergoing different training protocols. Although all training protocols improved at least one cardiometabolic parameter, the L-HIIT training protocol showed a significant impact on maximum oxygen consumption (VO2 max). This study suggests that the L-HIIT training protocol may potentially be a cost-effective means for T2DM patients to improve their quality of life. Likewise, Oberg et al. designed a randomized trial to evaluate the correlation between sleep patterns and endocrine parameters in over-weigh/obese women with polycystic ovary syndrome (PCOS). This study found that women with PCOS had poor sleep quality compared to the control group, suggesting the implementation of standard care with better sleep cycles to improve the endocrine and psychological well-being of women with PCOS. In addition, Gao et al. investigated homeostasis model assessment of insulin resistance (HOMA-IR) as a determining factor for metformin pre-treatment before *in vitro* fertilization/intracellular sperm injection (IVF/ICSI) and embryo transfer for patients with PCOS. This retrospective study found that metformin pre-treatment could improve pregnancy rates in women with PCOS with HOMA-IR during frozen IVF/ICSI-ET cycles. Díaz et al. delineated the influence of oral contraceptives (OCs) on endocrine-metabolic markers after 6 months of use of OCs in females with PCOS. This study showed that higher levels of circulating follistatin concentration correlated with insulin resistance and increased liver fat accumulation in females with PCOS compared to the control group. This indicates OCs treatment in females with PCOS may potentially cause adverse metabolic effects. Plessow et al. assessed the relationship between oxytocin levels and eating disorder in women with anorexia nervosa or atypical anorexia nervosa with primary food restriction (AN/AtypAN-R) and AN/AtypAN with restriction plus binge purge behaviors (AN/AtypAN-BP). This trial implies that both AN/AtypAN-R and AN/AtypAN-BP may be composed of fundamentally different neurobiology as they show distinct associations with eating, depressive, and anxiety parameters. Liu et al. analyze the association between serum total testosterone (TT) levels and metabolic syndrome (MetS) in women by logistic regression models. This study concludes that total TT levels in women are inversely correlated with MetS. However, future studies are needed to determine cut-off values for abnormal TT levels in women and their association with MetS at different scales. Wang et al. compared the difference in biochemical markers and degree of lesion visualization between primary hyperparathyroidism (PHPT) and secondary hyperparathyroidism (SHPT) by technetium 99m methoxyisobutylisonitrile (^99m^Tc-MIBI) imaging. This study revealed that the percentage of patients with positive dual-phase planar imaging on ^99m^Tc-MIBI was higher in the PHPT group compared to the SHPT group. The range of parathyroid lesions in SHPT was smaller than in PHPT. This study also suggests that a combination of other imaging technologies is needed if ^99m^Tc-MIBI imaging shows negative results in patents with SHPT for accurate lesion detection.

The inactivating follicle-stimulating hormone receptor (FSHR) genetic variants causes a wide spectrum of inconsistent clinical manifestations. Therefore, identification and molecular characterization of pathogenetic variants that disrupt FSHR protein function are important for the better diagnosis of primary ovarian insufficiency (POI) and resistant ovary syndrome (ROS). Using next-generation sequencing and traditional Sanger sequencing, Chen et al. identified rare compound heterozygous variants c.1384G>C/p.A462P and c.1862C>T/p.A621V in FSHR. Furthermore, *in vitro* characterization of these variants revealed a loss of intracellular signaling and receptor activation, respectively. This study expands our knowledge of understanding pathogenic variants of FSHR in connection to POI. Besides that, Tedjawirja et al. tested both monoclonal and polyclonal anti-FSHR antibodies on human premenopausal ovary, testis and skin samples and demonstrated that polyclonal anti-FSHR antibody non-specifically stained skin tissue, which is not known to express FSHR. This study points out the need for validated methods for better detection of FSH/FSHR in postmenopausal disease to avoid future challenges in the research field. Yu et al. characterized apolipoprotein C1 (APOC1) in diabetic nephropathy (DN) patients using functional gene enrichment analysis combined with mouse models and clinical samples. This study demonstrates that elevated serum and glomerular expression levels of APOC1 might be a novel biomarker for the diagnosis of DN. However, further research studies are needed to evaluate its diagnostic value. Refaat et al. investigated the prognostic value of simultaneous expression levels of estrogen receptor, progesterone receptor, and androgen receptors within the same cohort of colorectal cancer (CRC) patients in terms of gender, menopausal status, clinical stage, and tumor sidedness. The accurate determination of expression levels of sex steroid hormone receptors could significantly benefit the precise use of steroid blockers and hormonal therapy in CRC patients. Song et al. investigated the role of dihydrotestosterone (DHT) induced zinc transporter ZIP9 and its impact on learning, memory, and hippocampal synaptic plasticity of Tfm, APP/PS1 mice. This study revealed that ZIP9 facilitates the effects of DHT on hippocampal synaptic plasticity and dendritic spine density in Tfm mice via the ERK1/2-eIF4E pathway. Moreover, the same mechanism mediated by DHT can also affect learning and memory in APP/PS1 mice. Ziqubu et al. reviewed the influence of different parameters including diet, age, genetics, chemical exposure, and thermoneutrality on Brown adipose tissue/beige adipose tissue whitening and its relation to multiple metabolic complications including mitochondrial degeneration, mitochondrial dysfunction, autophagy, inflammation, devascularization and collapsed thermogenic capacity. Similarly, Fukunaga and Fujita reviewed the low glomerular number at birth and its relationship with the development of chronic kidney disease (CKD). This review expands our understanding of the significance of low glomerular number at birth increasing the risk of CKD and preventive prenatal nutritional management.

## Conclusion

Regulation of organ homeostasis is a complex process that involves the coordination of various biological mechanisms to maintain a stable internal environment within the body. The challenges lie in maintaining the delicate balance required for optimal organ function and preventing dysregulation that can lead to diseases. The knowledge gained from the Research Topic could be a steppingstone for future research to shed light on unresolved scientific problems and develop innovative treatment strategies for the life-threatening diseases.

## Future perspectives

The future perspectives in the regulation of organ homeostasis and the implications of hormones and immunity are exciting and hold tremendous potential for advancements in medicine. Precision medicine, immunomodulatory therapies, hormonal interventions for organ regeneration, modulation of signaling pathways, research on hormones and aging, innovative therapeutic delivery systems, and the integration of artificial intelligence based complex data analytics are among the areas that may shape the future of this field. Continued research and technological innovations will pave the way for more targeted and effective approaches to maintaining organ homeostasis and treating a wide range of life-threatening diseases.

## Author contributions

VKP wrote the original draft. All authors contributed to the editing equally and approved the submitted version.

